# A gender-related action of IFNbeta-therapy was found in multiple sclerosis

**DOI:** 10.1186/1479-5876-10-223

**Published:** 2012-11-14

**Authors:** Ida Contasta, Rocco Totaro, Patrizia Pellegrini, Tiziana Del Beato, Antonio Carolei, Anna Maria Berghella

**Affiliations:** 1 Consiglio Nazionale delle Ricerche (CNR), Istituto di Farmacologia Traslazionale (IFT), via G Carducci, 32-Rotilio Center, L’Aquila 67100, Italy; 2 Dipartimento di Neurologia dell’Università di L’Aquila, L’Aquila 67100, Italy

**Keywords:** Gender differences, Autoimmune diseases, Multiple sclerosis, Th cytokine networks, Th cell networks, CD30, Clinical targets, Clinical biomarkers

## Abstract

**Background:**

Understanding how sexual dimorphism affects the physiological and pathological responses of the immune system is of considerable clinical importance and could lead to new approaches in therapy. Sexual dimorphism has already been noted as an important factor in autoimmune diseases: the aim of this study was to establish whether sexual dimorphism in autoimmune diseases is the result of differing pathways being involved in the regulation of T-helper (Th) cell network homeostasis.

**Methods:**

We focused on sexually dimorphic changes in the immune response in multiple sclerosis (MS) patients in order to ascertain how these alterations relate to the pathway regulation of the cytokine homeostasis and the Th cell networks. We studied antigen presenting cell (APC)-dependent T cell activation in groups of healthy subjects, in patients under interferon (IFN) β-therapy and untreated. Cytokines, soluble (s) CD30 and the expanded disability status scale (EDSS) were used as biomarkers for T cell differentiation and neurological deficit.

**Results:**

The data confirm our belief that sexual dimorphism in autoimmune diseases is the result of differing pathways that regulate Th cell network homeostasis: interleukin (IL) 6 pathways in women and IFNγ pathways in men. Given the increased susceptibility of women to MS and the significance of IL6 in the autoimmune process compared to IFNγ, it is logical to assume that IL6 pathways are in some way implicated in the prevalence of autoimmune diseases in women. Indeed, our data indicate that IL6 pathways are also involved in T regulatory (Treg) cell imbalance and an increase in neurological deficit in both men and women groups of MS patients, underlining the autoimmune etiology of multiple sclerosis. In further support of differing cytokine pathways in men and women, we noted that the efficacy of IFNβ-treatment in the re-establishment of Th-network balance and in the delaying of the neurological disability progression is linked to the IL6 pathway in women, but to the IFNγ pathway in men. Lastly, we also identified specific gender biomarkers for the use in therapy.

**Conclusions:**

The identification of gender-specific drugs is of considerable importance in translational medicine and will undoubtedly lead to more appropriate therapeutic strategies and more successful treatment.

## Background

Sexual dimorphism in disease susceptibility is of considerable clinical importance and raises the question of gender specific drugs. The understanding of the physiological and pathological roles of sexual dimorphism in the immune response will clearly lead to improved gender-specific clinical therapy strategies. Autoimmune diseases highlight the need for further research in this field to better understand the reason why a large number of autoimmune diseases occur more frequently in women than men, such as MS. This female preponderance for abnormal autoimmune function has largely gone unexplained. There is evidence that sex hormones can affect the immune system and that female and male hormones act in opposing ways [1,2]. For example, Th1 and Th2 responses appear affected by androgenic and estrogenic preponderance, respectively: androgens favor the development of a Th1 response and the activation of CD8 cells [3], while estrogens seem to direct the immune system towards Th2 dominance, where B lymphocytes are activated and antibody production flourishes [2]. Pregnancy, a high estrogen state, is of course characterized by Th2 preponderance, and a failure in the establishment of the Th2 dominance has been associated with increased risk for pregnancy loss [4,5]. However, the basic reasons for the gender bias are not clear.

In the early 1980s, the Th1-Th2 hypothesis was put forward [6,7]: Th1 lymphocytes secrete pro-inflammatory cytokines (e.g. IL-2, IFNγ IL-12, and lymphotoxin) and they are specific against viruses and intracellular pathogens; whereas, Th2 cells secrete anti-inflammatory cytokines (e.g. IL-4, IL6, IL-5, IL-10) and act against extracellular pathogens, mediating humoral immune responses [7,8]. Subsequent research made it clear that the cytokine environment at the time of CD4+ T cell activation was the determining key in generating these effector subsets, due to the ability of cytokines to activate tailored transcription factors required for the differentiation of the specific Th subsets: Th1 requires the expression of T bet transcription factor; whereas, Th2 cells are controlled by expression of GATA-3 [9-11]. This concept led to the identification of other Th subsets with distinct functions in the immune response, namely Treg cells, Th17 cells and Th9 cells.

Treg cells produce the transforming growth factor (TGF) β cytokine, maintain Th cell network homeostasis and peripheral immunological tolerance. Forkhead box P3 (Foxp3), a member of the forkhead/winged-helix family of transcription factors, acts as a “master” regulator for the development and function of Treg cells and its constitutive expression is necessary for the specific role of Tregs. The mutation or deficiency of Foxp3+ Tregs cells is a key factor in the development of autoimmune diseases and the inability of the immune system to regulate the homeostasis in T-cell activation effectively [12,13].

Th17 cells secrete IL-17A and IL-17F, they arise in response to a combination of TGFβ plus IL6 pro-inflammatory cytokines and are controlled by the expression of the transcription factor retinoic acid-related orphan receptor gt (RORgt) [14-16]. The transcription factor of the more recently discovered Th9 subset [17,18], producing IL9 cytokine, is still to clarify. These cells develop from naive CD4 precursors in response to TGFβ plus IL-4 cytokines [17,18].

MS is an autoimmune inflammatory demyelinating disorder of the central nervous system. Gender influences both the susceptibility and the clinical course of MS: in fact the disease is more common in women than in men and there is an increased proportion of men developing the primary progressive form of MS [19]. The basis for these differences may include immunological factors but the exact mechanism has not yet been established.

There is a definite need for more effective biomarkers and therapeutic targets in MS. IFNβ therapy is only partially effective and not in all MS patients. IFNβ therapy can reduce the severity and frequency of relapses, disease activity (as measured by magnetic resonance imaging) [20,21], and disability (as measured by EDSS scores) by inducing mechanisms that determine a fall in sCD30 levels and a re-establishment of homeostasis in the Th-cell network [22-24].

On the basis of the above we investigated the possibility that sexual dimorphism in autoimmune diseases could depend on sexual dimorphism in the regulation of Th cell network homeostasis. We carried out studies using the peripheral blood of healthy subjects, IFNβ-treated and untreated relapsing remitting (RR) MS patients, as independent cohorts. We used systems biology to study APC-dependent T cell activation. Cytokines, sCD30 and EDSS parameters, biomarkers of direction in T cell differentiation and neurological deficit, were used to relate male and female gender to the cytokine pathways regulation of Th cell differentiation.

## Methods

### Experimental design

In physiological systems the different components operate as a network: they vary dynamically and co-vary each other respectively. Therefore, the identification of physiological pathways can only be achieved through evaluations of the systems biology characteristics. Due to the complexity of biological systems, this process requires the use of mathematical models that provide a framework for determining the outcome of numerous and simultaneous time-dependent and space-dependent processes [25-27].

We designed an experimental approach based on the use of cytokine data-driven models of the immune response. All persons gave their informed consent prior to their inclusion in the study.

We used the levels of Th cytokines produced by APC (IFNy, IL6, IL10) as biomarkers of Th cell differentiation: the relative proportion of each Th cell-type generation depends, in fact, on the relative proportion of specific cytokines produced by APCs and released into the cell environment during resting and activation states of the immune response.

To investigate whether sexual dimorphism of Th cytokine pathways and regulation of Th cell network homeostasis are normally present in the immune response, we determined the profiles of the levels of Th cytokines in the supernatants of 72 h whole blood cultures without activation (APC and T cells in resting conditions), with lipopolysaccharide (LPS) (activated APCs) and phytohaemagglutinin (PHA) (activated T cells) stimuli, in healthy subjects. We also analyzed the PHA-level of cytokine network profiles in supernatants of 24 h culture, separated (Ficoll/Hypaque gradient) peripheral blood mononuclear cells (PBMC), in order to discover how T cellular components affect the cell network interactions.

To establish whether sexual dimorphism in autoimmune diseases depends on differences in the pathways responsible for the regulation of Th cell network homeostasis, we determined, in treated and untreated RRMS patients, Th cytokine profiles in culture supernatants of: i) immature (I) dendritic cells (DC), to study the APC regulation of Th pathways in immune resting conditions; ii) DC after LPS stimulus, to study APC regulation of Th pathways in activated conditions; iii) whole blood without activation (APC and T cells in resting conditions), and after PHA stimulus (activated T cells), in order to clarify APC and Th cell interaction.

The cytokines used as biomarkers in the immune response models were: IL2, IL12p70, IFNy, IL4, IL12p40, IL6, IL10 and TGFβ. IL2, IL12p70 and IFNy support Th1 functions [28] promoting cell mediated immunity; IL12p40, IL4, IL6 and IL10 are associated with Th2 responses and IL10 is a powerful inhibitor of IFNγ and macrophages [29]. TGFβ together with IL6 also supports Th17 functions (suppressing Th1 functions) [30,31], whereas TGFβ together with IL4 supports Th9 functions [32]; IFNy inhibits Th9 functions [32].

sCD30 was used as a biomarker for homeostasis or alteration in immunological and neurological pathways to evaluate the success or failure treatment. In fact, our recent results indicate that the up- and down-regulation of sCD30 levels within physiological ranges is a biomarker for homeostasis in immunological and neurological pathways and therapeutic success. However a significant increase in sCD30 levels suggests immunological alterations, neurological deficit and treatment failure [22]. Our results underlined that sCD30 pathways control immunological and neurological system homeostasis by regulating the type of Th differentiation through the cytokine profiles of IDCs and DCs, which are major professional APCs that initiate and modulate immune response by inducing Th0 differentiation [22].

### Healthy subjects

A group of 66 healthy subjects (33 men and 33 women: blood donors, laboratory staff and relative) were studied. None of the subjects was receiving concurrent drug treatment including widely-used pharmaceuticals, such as salicylates and sex hormones (contraceptive pill, hormone replacement therapy). Distribution of age in male and female groups was the same (mean ± SD = 41±12 years, compared to mean ± SD = 41 ± 15 years, p = 0.14).

### Independent validation cohorts of multiple sclerosis patients

We investigated 36 patients with clinically defined RRMS, admitted to the MS Center of the Department of Neurology of the University of L’Aquila. 18 of these patients were receiving IFNβ-therapy (the treated group) and 18 were not (the untreated group). None of the patients had suffered an exacerbation in the 3 months before entry into the study or received corticosteroids in EDSS. Neurological examinations were carried out by neurologists from the MS Center of the Department of Neurology of the University of L’Aquila. EDSS was used to assess patient neurological deficit as MS is a chronic, lifelong disease and long term deficit data need to be evaluated. We considered the absence of clinical relapses of at least 3 months, a marker of disease inactivity. The treated group consisted of 5 men and 13 women, with a mean age ± SE of 34.3 ± 2.2 and of 31 ± 1.8 years respectively; and a mean EDSS score ± SE of 1.8 ± 0.6 and 2.2±0.3 respectively; All had been receiving IFNβ1 a treatment [22 μg (4 patients, 1 men and 3 women) or 44 μg (14 patients, 4 men and 10 women] subcutaneously thrice weekly for a mean period±SE of 27.5 ± 1.4 months (range: 2 to 60 months]. The untreated group of patients, matched for sex (p = 0.6), age (p = 0.3) and EDSS (p = 0.9) score, consisted of 7 men and 11 women with a mean age±SE of 37.2 ± 4.7 and of 32 ± 9.1 respectively; and a mean EDSS score±SE of 1.9 ± 0.4 and of 2 ± 0.3 respectively. Distribution of age, EDSS, treatment dose and period of treatment in male and female groups was the same not only within, but also between the groups of treated and untreated patients. This was a paired samples study: all parameters were determined for each patient sample.

### Blood samples

Blood was collected at the same time of day to minimize the effects of diurnal variation.

### Whole blood cell cultures

The whole blood culture method [33] was used since it reflects in vivo physiological conditions more accurately [34]. Briefly, a 15 ml sample of heparinized blood (20 IU heparin/ml blood Liquemin Roche) was taken from each subject, and the samples, kept at room temperature, were used immediately. Venous blood was diluted 1:10 with RPMI 1640 medium (Sigma, endotoxin tested), which was supplemented with 0.2 mM of L glutamine, 50 IU/ml of penicillin, 50 μg/ml of streptomycin (Sigma) and 10% of heat-inactivated serum. Aliquots at 1 × 10^6 ^cells were distributed in 12 mm polypropylene tubes. 10 μg/ml of PHA (Sigma) and 10 μg/ml of LPS (Sigma) were used for stimulation and aliquots without stimuli were also prepared. After 3 days of culture the supernatant was removed to be assayed for cytokine levels and stored in aliquots at −80°C until used. The effect of adding heparin, which prevents clotting in whole blood cultures, was tested (data not shown). Experimental conditions were: with stimuli (+PHA and +LPS) in order to re-create an activation situation and without stimuli, to evaluate immune response in resting conditions. PHA was used to study T cell contribution [35] and LPS to evaluate the influence of antigen presenting cells [36].

### PBMC cell cultures

PBMC were separated by centrifugation over a Ficoll/Hypaque gradient (20 min, 1000 g), and washed with RPMI-1640 medium (Gibco). Isolated cells were cultured at a concentration of 1 × 10^6 ^cells/ml in RPMI-1640 complete medium (supplemented with 10% of fetal calf serum, L-glutamine 0.2 nM, penicillin 50 UI/ml, streptomycin 50 g/ml; Sigma). Supernatants were obtained from PBMC cultures in RPMI-1640 complete medium. The cells (with and without: PHA, 3 g/ml or LPS, 10 g/ml) were incubated at concentrations of 1×10^6 ^cells/ml at 37°C in a humidified atmosphere of 5% CO2. After 24 h of culture without a change of medium, supernatant was removed from each well, centrifuged at 250 g and stored in aliquots at −80°C until use.

### Generation of DCs from monocytes

A sample of heparinized blood (20 IU/ml blood - Liquemin-Roche) was taken from each subject and diluted 1:4 with medium at room temperature. PBMCs were separated by centrifugation (160 g for 20 minutes at 20°C) over a Lymphoprep gradient (Nycomed Norvay). PBMCs were recovered from plasma/Lymphoprep interface, washed three times with medium (200 g for 10 minutes at 20°C).

Monocytes from PBMC samples were obtained by removing T cells, B cells, NK cells and granulocytes (if present): antibody mix was added to the PBMC sample and then depletion Dynabeads were added to capture the antibody bound cells. Dynabeads are uniform, supermagnetic, polystyrene beads coated with a Fc specific human IgG4 antibody against mouse IgG. The antibody mix contained a mixture of mouse monoclonal antibody for CD2, CD7, CD16 (specific for CD16a and CD16b), CD19 and CD56. The blocking reagent was gamma globulin to block Fc receptors (FcR) on monocytes. These coated cells were then separated with a magnet (Dynal MPC) and discarded. PBMCs were then transferred into a 15 ml tube, washed three times with PBS by centrifugation (at 225 g for 8 minutes at 2-8°C) and then re-suspended at 1 × 10^7 ^per 100–200 μl PBS. 22 μl of blocking reagent and 20 μl of antibody mix for each 1 × 10^7 ^MNC cells were added and incubated for 10 minutes at 2-8°C. The cells were washed (by adding 1 ml of PBS per 1 × 10^7 ^MNC and centrifugation for 8 minutes at 500 g) and re-suspended in 0.9 ml of PBS per 1 × 10^7 ^MNC. 100 μl of Depletion Dynabeads per 1 × 10^7 ^MNC was added and incubated for 15 minutes at 2-8°C with gentle tilting and rotation. The total volume for cell and bead incubation should be 1 ml per 1 × 10^7 ^MNC. The rosettes were re-suspended by careful pipetting 5–6 times before increasing the volume by adding 1–2 ml of PBS and placed in the Dynal Magnetic particle concentrator for 2 minutes. Isolated monocytes recovered were > 98% viable and free of surface bound antibody or Dynabeads. Monocytes were re-suspended in RPMI 1640 (Sigma, endotoxin tested) complete medium containing 10% of AB male human serum (heat-inactivated for 30 minutes at 56°C), penicillin (50 UI/ml), streptomycin (50 μg/ml) and L-glutamine (0.2 mM).

The cells were incubated in 24-well plates at a concentration of 200,000 cells per ml at 37°C in a humidified atmosphere of 5% CO2 for 8 days and stimulated with 30 ng/ml of recombinant IL4 (Preprotech-England, specific activity of > 5 × 106 U/mg), 30 ng/ml of recombinant GM-CSF (Preprotech-England, specific activity of > 1 × 107 U/mg). On day 3, IL4 and GM-CSF were added again and on day 6, immature DCs were obtained. Immature DCs were stimulated with 100 μg/ml of LPS (Sigma), and DCs without stimuli were also prepared. After 2 days of culture the supernatant was removed from each well, centrifuged at 250 g and stored frozen in aliquots at ¬80°C until used.

### Cytokine detection

Cytokine levels and soluble molecules were determined using a solid phase sandwich ELISA method [37]. For intra assay precision, samples of known cytokine concentration were assayed in replicates of 10 (to determine precision within an assay) and the coefficient of variation is < 10% (data not shown). For inter assay precision, samples were assayed 30 times in multiple assays (to determine precision between assays), the coefficient of variation was < 10% (data not shown). The ELISA assay sensitivity was: IL10 < 1 pg/ml, TGFβ1< 1.9 pg/ml, IL2 < 10pg/ml, IFNγ< 5 pg/ml, IL6 < 2 pg/ml, (Euroclone), IL12p40 < 15pg/ml, IL12p70 < 0.5 pg/ml, IL4<0.13 pg/ml (R&D System), sCD30 < 0.5 U/ml, sBcl2 < 1 U/ml (Bender Medsystem).

### Data analysis

In physiological systems components operate as a network and each component varies and co-varies dynamically with respect to one another. Therefore, the identification of physiological pathways, and correlated biomarkers can only be achieved through evaluations that take these fluctuations into account. Hence we used systems biology studies [38,39] which allowed us to analyze the relationships between parameters (levels of cytokines, sCD30 and EDDS values) and the behaviour of this multicomponent system as a network. Thus, in addition to the study of statistical differences using the Mann Whitney U test or Student’s t test as appropriate, we used multivariate statistical analyses [25-27,40] by “Statgraphics software systems” (full system 5.25 version 4.0”- Graphics system by statistical graphics corporation ed. USA. 1989). Values of p ≤ 0.05 were considered significant.

We used the multivariate statistical procedure that analyses the correlation between parameters and produces a matrix of correlation coefficients (that vary from −1 to +1) and significance (p), allowing a dynamic analysis of how network components vary with respect to one another at any moment in time. A positive correlation indicates that the parameters vary in the same direction, while negative correlation indicates that the parameters vary in the opposite direction. In fact, the multivariate statistical procedure that analyses the correlation measures the linear associations between all parameters; if parameters increase or decrease at the same time the correlation is positive, while other changes are considered negative. Statistically independent parameters have an expected correlation of zero.

**Figure 1:**
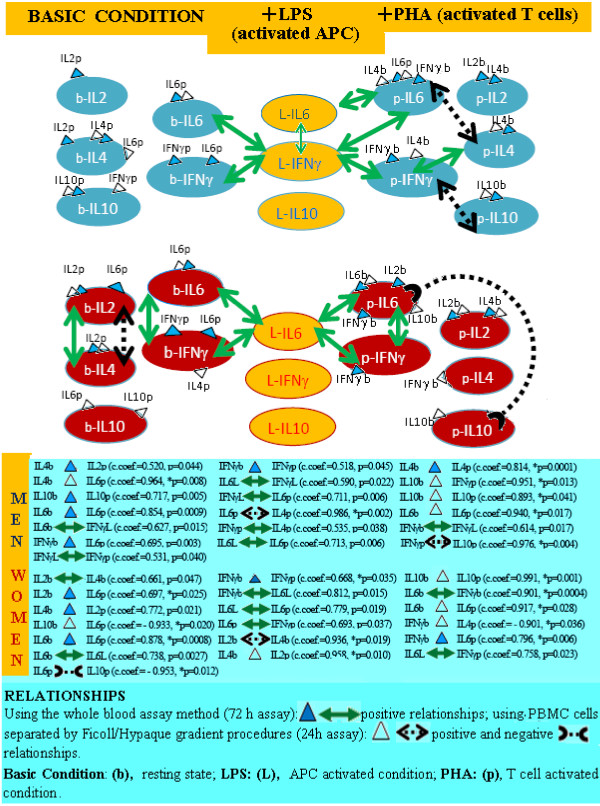
**APCs regulate Th cell differentiation and Th cell network homeostasis in both men and women, and this effect appears to be exerted through IFNγ production in men, but through IL6 production in women.** No significant relationships were observed in either the 24 h (early regulation) or 72 h (late regulation) cultures in resting conditions (b-cytokines) in men. In women, relationships were found between production of IL2-IL4 and IFNγ-IL6 Th1/Th2 cytokines in resting conditions (b-cytokines). The IL2 and IL4 inter-regulation in women was significant in both the 24 h and 72 h culture supernatant cytokine assays, while the inter-regulation between IL6 and IFNγ levels is only significant in the 72 h assay. Significant relationships emerged in cell cytokine production in activated (+PHA ) conditions in both, men and women. In the 24 h (early regulation) PHA culture: positive linked production between IL6 and IL4, and IFNγ and IL10 cytokines (p-cytokines) were found in men, and negative linked production between IL6 and IL10 cytokines (p-cytokines) were found in women. In the 72 h (late regulation) PHA culture: positive linked production between IFNγ and IL4 were found in men, while positive linked production between IFNγ and IL6 were found in women (p-cytokines).

Using the principal component analysis (PCA) we plotted the network of vectors obtained by analyzing the data matrix of correlation coefficients (Figure 1). In the plots obtained, the angle between vectors is inversely proportional to the degree of correlation between vectors; the same vector direction indicates a positive correlation/covariance, the opposite vector direction indicates a negative correlation/covariance. This allows a visualization of the situation under study and is an excellent method for capturing significance from systems biology evaluations [41].

## Results

### In health, APCs regulate Th cell differentiation and Th cell network homeostasis through IL6 pathways in women but through IFNγ pathways in men

Our results confirm that in health, cytokine regulate immune response cell phases through gender specific pathways.

No significant differences were observed in the levels of the different types of specific Th cytokines between men and women apart from IL10, which was higher in men when PHA stimulus was used (Table 1).

**Table 1:** The levels of the different types of specific cytokines in healthy subjects

**Whole blood and PBMC supernatants**					
**Healthy subjects**					
**Men**					
**Basic condition**			**PHA**		**LPS**
**pg/ml**	**Mean**^**wb **^**± SD**	**Mean**^**s **^**± SD**	**Mean**^**wb **^**± SD**	**Mean**^**s **^**± SD**	**Mean**^**wb **^**± SD**
IL10	4,9 ± 13,4	26 ± 40,2	37,6 ± 43,2	93,7 ± 116,7	61,1 ± 96,4
IFNγ	149 ± 194,3	161,8 ± 146,2	1752 ± 2344,5	415,7 ± 389,3	1473,2 ± 2408
IL6	133,3 ± 376,2	390,7 ± 495,7	235,7 ± 303,2	1386,3 ± 2138,3	573,5 ± 714,9
IL2	256 ± 181.8	64,1 ± 78,2	248,7 ± 233,4	290,4 ± 196,8	
IL4	15,5 ± 32,2	7,4 ± 10,4	21,9 ± 29	21,8 ± 24,4	
**Women**					
**Basic condition**			**PHA**		**LPS**
**pg/ml**	**Mean**^**wb **^**± SD**	**Mean**^**s **^**± SD**	**Mean**^**wb **^**± SD**	**Mean**^**s **^**± SD**	**Mean**^**wb **^**± SD**
IL10	26 ± 46,3	155 ± 180,8	6,9 ± 8,8	143 ± 155,4	72,9 ± 150,3
IFNγ	70,1 ± 110	173,2 ± 74,4	1300,3 ± 2294	1071,1 ± 1102,2	1603 ± 4603
IL6	34,8 ± 67,9	243,3 ± 293,4	104,4 ± 156	1921,5 ± 1654	316,7 ± 322,6
IL2	153,6 ± 140	238,8 ± 247,1	222,4 ± 193	573,4 ± 438,1	
IL4	29,4 ± 47,7	24,3 ± 36,6	76,6 ± 115,7	9,7 ± 4,7	

Cytokine levels were determined in whole blood (wb) and PBMC supernatant (s) in resting (Basic conditions) and activation conditions after stimulation with PHA (to study T cell network contribution) and LPS (to evaluate the influence of antigen presenting cells). The levels are expressed as mean ± SD, and statistical differences between men and women were assessed using the Mann–Whitney U-test or Student’s t-test as appropriate. Production levels of cytokines did not differ significantly with the exception of IL10 (using the ^wb ^method) which was higher in men than in women when PHA stimulus was used (p = 0.038).

Our results show that APCs regulate Th cell differentiation and Th cell network homeostasis under resting and activated conditions of the immune system in both men and women: relationships were found between APC cytokines (L-cytokines: IFNγ, L6, IL10) and Th cell cytokines under resting (b-cytokines: IL2, IL4, IFNγ, L6, IL10) and activated (p-cytokines: IL2, IL4, IFNγ L6, IL10) conditions (Figure 1). However, this effect appears to be exerted through IFNγ production in men but through IL6 production in women (Figure 1).

The production of cytokines by cells in resting conditions does not seem to have a specific role in the regulation of Th cells in men (Figure 1, men b-cytokines), since no significant relationships were observed in either the 24 h (early regulation) or 72 h culture (late regulation). However in women the linked production of IL2-IL4 and IFNγ-IL6 Th1/Th2 cytokines appears to influence regulation (Figure 1, women b-cytokines). Furthermore, this Th1/Th2 IL2 and IL4 inter-regulation in women appears to have an early and late role in the control of the Th-cell network since the correlation between their levels is significant in both the 24 h and 72 h culture supernatant cytokine assays. The inter-regulation between IL6 and IFNγ levels is only significant in the 72 h assay (cytokine assay of the whole blood supernatant after 72 h of cell culture).

Cell cytokine production in activated conditions appears to have a specific role in the regulation of Th cell network homeostasis in men and women as significant relationships emerged. Figure 1 shows that the early evolution of activated T cells (24 h, +PHA culture) is influenced by the positive linked production of IFNγ and IL10, and IL6 and IL4 cytokines in men, and the negative linked production of IL6 and IL10 cytokines in women (Figure 1, p-cytokines). Similarly, the late evolution of activated T cells (72 h, +PHA culture) seems to be influenced by the positive linked production of IFNγ and IL4 in men while by IFNγ and IL6 in women (Figure 1, p-cytokines).

**Figure 2:**
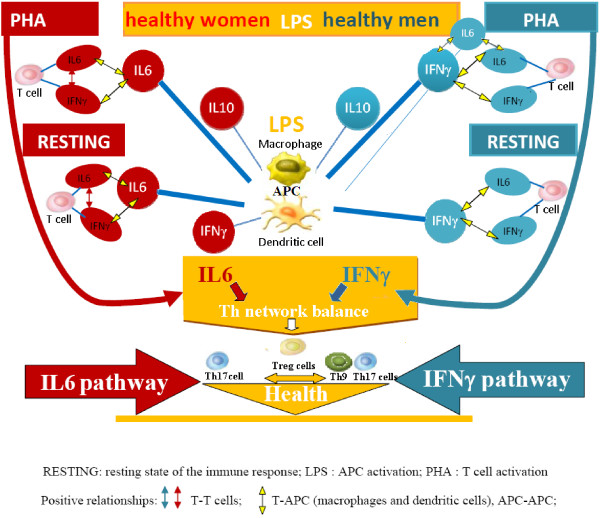
**Gender dimorphism of Th cytokine pathways and regulation of Th cell network homeostasis are normally present in the immune response.** The multivariate statistical analysis of the correlation between the levels of Th cytokines (Table 1) confirms the cytokine regulation of immune response cell phases through gender specific pathways. APC cytokines regulate the resting and activated (PHA) cell phases of immune response, through gender specific pathways: IL6 pathways is the gender specific pathways in women, but IFNγ pathways is the gender specific pathways in in men. Consequently, autoimmune disease susceptibility in women could be attributed to the influence of ΙL6, since IL6 prevents the conversion of naive Th into Treg cells in vivo, by switching Th cell differentiation from Treg to Th17, which plays a key role in autoimmune diseases. The greater probability of men developing the primary progressive form, conversely, could be the result of the influence of IFNγ on Th9 cell inhibition, since co-expression of IL-9 and IL-17 was identified as a novel Th17 function in mediating autoimmune tissue destruction, including the CNS.

These results therefore confirm cytokine regulation of immune response cell phases through gender specific pathways (Figure 2). Hence, autoimmune disease susceptibility in women could be attributed to the influence of ΙL6, which plays a key role in autoimmune diseases, since it is a T cell differentiation switch factor from Tregs to Th17 cells [14-16]. The greater likelihood of men developing the primary progressive form, on the other hand, could be the results of the influence of IFNγ on Th9 cell inhibition [42] (Figure 2).

Clearly, different gender pathways result in differing alterations in T cell differentiation and consequently the generation of different pathological mechanisms and diseases in men and women.

### IL6 pathways are involved in Th cell network imbalance and an increase in neurological deficit in both men and women suffering from MS

Furthermore, we found that the susceptibility of the female sex to abnormal autoimmune function can be attributed to the dominant role of ΙL6 in Tregs imbalance.

**Figure 3:**
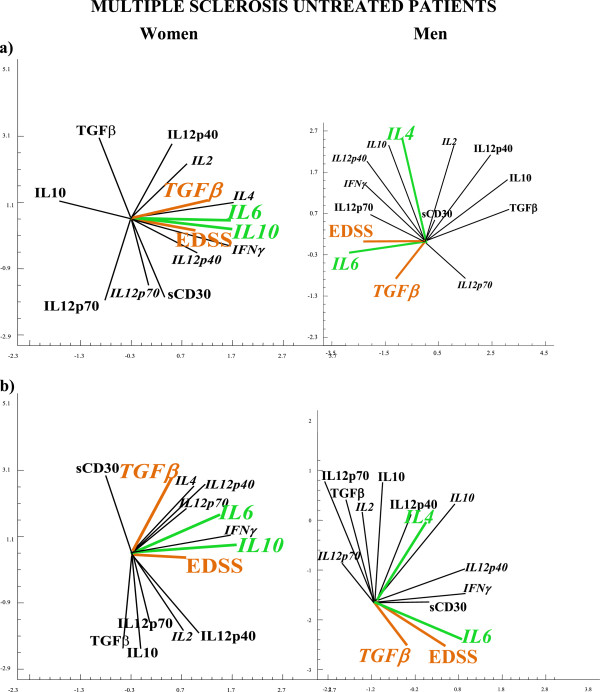
**APC analysis in MS untreated patients using IDC, DC and whole blood Th-cytokine data-driven computational models of disease state.** Independent cohorts of MS untreated patients were evaluated, using IDC, DC and whole blood Th-cytokine data-driven computational models of disease state. We used the multivariate statistical procedure of principal component analysis (PCA) which allowed us to analyze the relationships between parameters (levels of cytokines, sCD30 and EDDS values) and the behaviour of this multicomponent system as a network. The levels of cytokines were used as biomarkers of Th differentiation (Treg, Th1, Th9 or Th17) and EDSS was used as biomarker for neurological deficit. Using the PCA analysis we plotted the network of vectors obtained by analyzing the data matrix of correlation coefficients. In these plots, the angle between vectors is inversely proportional to the degree of correlation between vectors: the same vector direction indicates a positive correlation/covariance, the opposite vector direction indicates a negative correlation/covariance. This allows a visualization of the situation under study and our results indicate that the IL6 pathway is involved in neurological deficit increase and Treg imbalance in resting (Figure 3a) and activated (Figure 3b) conditions in untreated, male and female patient groups. This we concluded from the fact that cytokine level increases of IDC/DC IL6 and TGFβ (the dual biomarker for an increase in Th17 cells and a fall in Treg cells) were positively related to increases in neurological deficit (EDSS) (Figures 3a and 3b).

**Figure 4:**
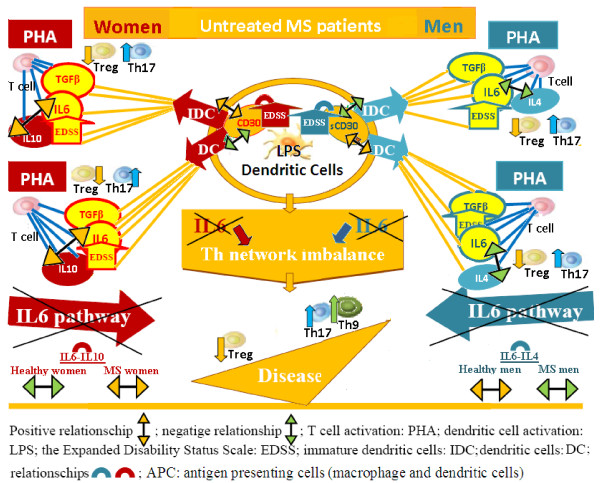
**The IL6 pathway alteration is involved in neurological deficit increase and Tregs imbalance in untreated male and female patient groups, underlining the autoimmune etiology of multiple sclerosis.** Immunological alterations in IL6 pathways are responsible for the Treg imbalance and increase of EDSS in both sexes in untreated MS patients. In fact, IL6 together with TGFβ pathways, a dual biomarker for Treg/Th17-network imbalance, were involved in the EDSS increase in both untreated, men and women groups of MS patients (Figures 3c and 3d: the vectors of TGFβ IL6 and EDSS are positively related). Furthermore, in health, APC IL6 pathways regulate Th cell differentiation and Th cell network homeostasis by the positive linked production of IL6 and IL4 in men, and the negative linked production of IL6 and IL10 cytokines in women (Figure 1, p-cytokines). Whereas in untreated MS patients, the relationship between the production of IL6 and IL4 had changed from positive to negative in men, while the relationship between the production of IL6 and IL10 had changed from negative to positive in women (Figures 3c and 3d).

**Figure 5:**
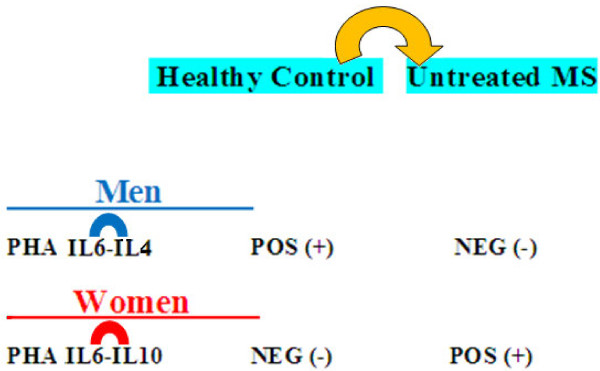
**IL6 pathways regulate Th cell differentiation and Th cell network homeostasis in healthy men and women.** In health APC IL6 pathways regulate Th cell differentiation and Th cell network homeostasis by the positive linked production of IL6 and IL4 in men, and the negative linked production of IL6 and IL10 cytokines in women. Instead (Figures 3 and 4), in MS untreated patients the relationship between the production of IL6 and IL4 changes from positive to negative in men, while the relationship between the production of IL6 and IL10 changes from negative to positive in women.

To establish whether sexual dimorphism in MS is the result of sexual dimorphism in the regulation of Th cell network homeostasis in the immune response, independent cohorts of MS patients (treated and untreated) were evaluated, using IDC, DC and whole blood Th-cytokine data-driven computational models of disease state. Our results (Tables 2 and 3) indicate that the IL6 pathway alteration is involved in neurological deficit increase and Tregs imbalance in resting (Figure 3a) and activated (Figure 3b) conditions in untreated male and female patient groups, underlining the autoimmune etiology of multiple sclerosis (Figure 4). This we concluded from the fact that cytokine increased levels of IDC/DC IL6 and TGFβ (the dual biomarker for an increase in Th17 cells and a fall in Treg cells) were positively related to increases in neurological deficit (EDSS) (Figures: 3a, 3b and 4). Additionally, as mentioned previously (Figure 1, p-cytokines), we found that APC IL6 pathways regulate Th cell differentiation and Th cell network homeostasis by the positive linked production of IL6 and IL4 in healthy men, and the negative linked production of IL6 and IL10 cytokines in healthy women. In untreated MS patients, on the other hand, we found that the relationship in the production of IL6 and IL4 in men had changed from positive to negative while the relationship in the production of IL6 and IL10 in women had changed from negative to positive (Figures 3, 4 and 5).

**Table 2:** The levels of cytokines in culture supernatants of IDC and DC in MS patients

**Cytokines(pg/ml**^**∘**^**U/ml**	**Treated patients**			
	**IDC**		**IDC-LPS**	
	**Man**	**Women**	**Man**	**Women**
IL10	5.3 ± 0.1**a**	3.3 ± 0.9	73.9 ± 37.5	41.2 ± 7.9
IL12p70	0 ± 0.02	0.13 ± 0.13**b**	3.3 ± 3.1	4.5 ± 1.3**i**
IL12p40	58.2 ± 11.4	35.8 ± 5.9**d**	3834 ± 796**l**	3863 ± 260**m**
TGFβ	22156 ± 1091**e**	21792 ± 701**f**	20701 ± 1103**n**	20164 ± 633**p**
^∘^sCD30	7.01 ± 0.4**g**	6.9 ± 1.1**h**	13.5 ± 1.1**q**	14.7 ± 1.1**r**
**Cytokines(pg/ml**^**∘**^**U/ml**	**Untreated patients**			
	**IDC**		**IDC-LPS**	
	**Man**	**Women**	**Man**	**Women**
IL10	0.6 ± 0.6**a**	1.1 ± 0.5	27.5 ± 19.2	55.8 ± 13.1
IL12p70	0.37 ± 0.9**c**	2.9 ± 0.6**b,c**	9.7q ± 6.5	17.1 ± 3.8**i**
IL12p40	51.9 ± 6.8	63.7 ± 7.3**d**	5999 ± 277**l**	5094 ± 647**m**
TGFβ	16582 ± 813**e**	16400 ± 575**f**	14726 ± 642**n**	14210 ± 601**p**
^∘^sCD30	27.2 ± 24**g**	26.8 ± 5**h**	36.9 ± 3.2**q**	38.1 ± 3.5**r**

Cytokine levels were determined in culture supernatants of IDC (to evaluate the APC influence on immune response in resting conditions) and of DC (to evaluate the APC influence on immune response in activation conditions). The levels are expressed as mean (or median) ± standard error, as appropriate. Statistical differences between groups were determined using the Mann–Whitney U-test or Student’s t-test as appropriate: **aa** p = 0.008; **bb** p < 0.0001; **cc** p = 0.03; **dd** p = 0.006; **ee** p = 0.004; **ff** p < 0.0001; **gg** p = 0.0007; **hh** p < 0.0001; **ii** p = 0.001; **ll** p = 0.013; **mm** p = 0.006; **nn** p = 0.001; **pp** p = 0.001; **qq** p = 0.001; **rr** p = 0.001.

**Table 3:** The levels of cytokines in whole blood culture supernatants in MS patients

	**Treated Patients**		**Untreated Patients**	
**Cytokines(pg/ml)**	**Basic condition**		**Basic condition**	
	**Men**	**Women**	**Men**	**Women**
IL10	2.13 ± 0.4	1.8 ± 0.2	1.5 ± 0.1	1.8 ± 0.2
IFNγ	16.1 ± 5	11.4 ± 3.2	14 ± 2.3	19.8 ± 3.6
IL6	232 ± 227**a**	10 ± 9.8**a**	35.8 ± 11.5	12.0 ± 17.5
IL2	10.8 ± 1	8.7 ± 0.9	8.7 ± 3.5	9.4 ± 1.5
IL4	1.1 ± 0.2	1.1 ± 0.1	0.7 ± 0.1	0.7 ± 0.1
IL12p70	9.2 ± 0.9	9.5 ± 0.8	11.2 ± 1.2	8.4 ± 0.7
IL12p40	25.2 ± 2.6	21.6 ± 3	20.1 ± 6.5	20.7 ± 5.1
TGFβ	7324 ± 833	5903 ± 458	10721 ± 5822	5758 ± 386
	**PHA**		**PHA**	
	**Men**	**Women**	**Men**	**Women**
IL10	59.7 ± 24.9	23.7 ± 11.8	78 ± 17.4	49.4 ± 30.2
IFNγ	1149.2 ± 570	740 ± 112.9**b**	2238 ± 904	2102 ± 658**b**
IL6	759 ± 359	627 ± 109.1	673 ± 427	648.1 ± 153.8
IL2	18.3 ± 5.9	13.9 ± 7.4	20.6 ± 8	21.3 ± 4.1
IL4	9.7 ± 5.3	3.6 ± 1.3	3.6 ± 1.1	1.2 ± 1.5
IL12p70	13.4 ± 2.5	9.9 ± 0.6	9.1 ± 1.5	9.3 ± 0.9
IL12p40	196 ± 105	101.7 ± 15.4	186 ± 82	86.2 ± 15.4
TGFβ	7644 ± 906	6953 ± 583	16029 ± 8457	8612 ± 4630.1
	**LPS**		**LPS**	
	**Men**	**Women**	**Men**	**Women**
IL10	144 ± 28.2	215 ± 54.7	181 ± 50	195.5 ± 48.6
IFNγ	135 ± 37.9	71.2 ± 139.4	1131 ± 809.3	263 ± 88.5
IL6	989 ± 89	635.9 ± 112.5	795 ± 173	813.2 ± 123.7
IL2	15.6 ± 3.7**c**	11 ± 4.4	7.6 ± 1.5**c**	12 ± 1.6
IL4	1.54 ± 0.5	1.1 ± 0.1**d**	0.7 ± 0.1	0.7 ± 0.06**d**
IL12p70	11.5 ± 1.9	8.9 ± 0.8	9.7 ± 0.9	9.1 ± 0.7
IL12p40	416 ± 309	354.4 ± 69.5	1203 ± 664	284.2 ± 87.2
TGFβ	7582 ± 832	7524.6 ± 3492.7**e**	12669 ± 6570	16984 ± 4854.8**e**

Levels were determined by whole blood assays in resting conditions (Basic conditions) and after stimulation with PHA (to principally study the activated T cell network contribution) and LPS (to evaluate the influence of activated antigen presenting cells). The level values are expressed as mean ± SD and statistical differences between groups were assessed using the Mann–Whitney U-test or Student’s t-test as appropriate: **aa** p = 0.027; **bb** p = 0.023; **cc** p = 0.039; **dd** p = 0.002; **ee** p = 0.031.

### Further confirmation comes from the fact that IL6 pathways in women and IFNγ pathways in men, are responsible for the efficacy of IFNβ-therapy in re-establishing Th network balance and delaying the progression of neurological disability in treated patients

Then again, the IL6 pathway in women but the IFNγ pathway in men were responsible for delaying EDSS.

**Figure 6:**
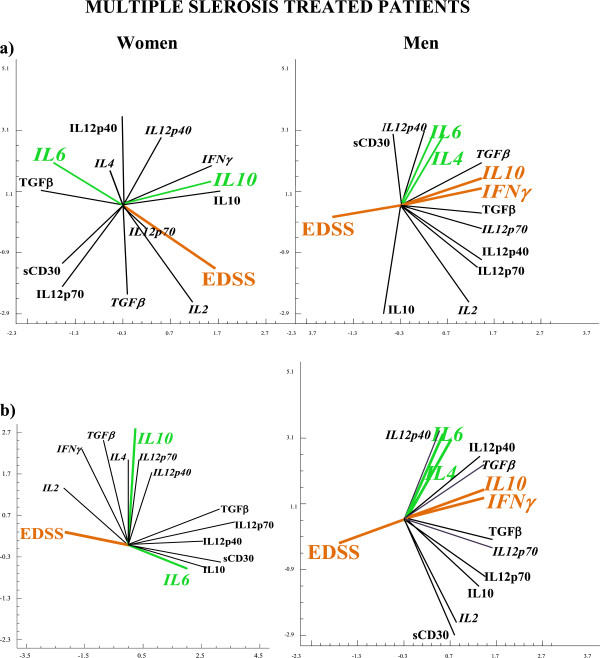
**APC analysis in MS treated patients using IDC, DC and whole blood Th-cytokine data-driven computational models of disease state.** Re-establishment of IL6 immunological pathways are responsible for the re-establishment of neurological (no increase of EDSS) and immunological (Th cell-network) homeostasis in treated patients and is induced by gender specific cytokine pathways. In fact , the IL6 vector in women, and the IFNγ vector in men, negatively opposed their respective EDSS vectors and the relationships between the production of IL6, IL4 and IL6, IL10 reverses to that found in healthy subjects, in both sexes.

**Figure 7:**
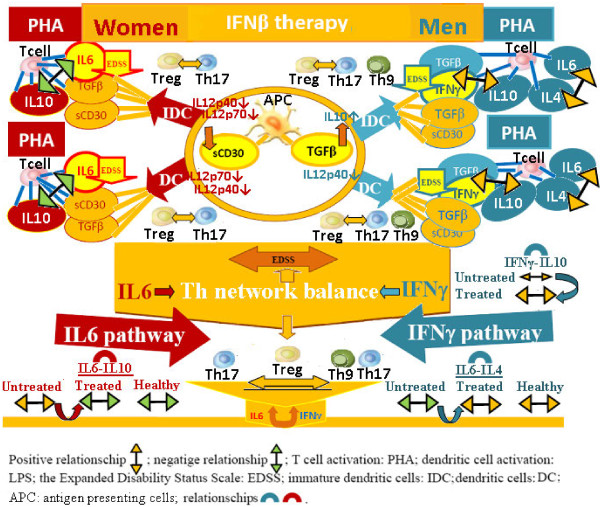
**The re-establishment of immunological (Th cell-network) and neurological (no increase of EDSS) homeostasis in treated patient is induced by gender specific cytokine pathways.** The results confirm cytokine regulation of immune response cell phases through gender specific pathways. Autoimmune disease susceptibility in women could be attributed to the influence of ΙL6, which plays a key role in autoimmune diseases, since it is a T cell differentiation switch factor from T-regs to Th17 cells. The greater likelihood of men developing the primary progressive form, on the other hand, could be the results of the influence of IFNγ on Th9 cell inhibition. Targets and biomarkers for clinical monitoring and translational research for the development of gender specific therapy, were found. sCD30 -TGFβ is a dual clinical biomarker for both sexes, while IL6-IL10 is a dual clinical biomarker specific for women and IL6-IL4 together with IFNγ-IL10 are dual clinical biomarkers specific for men. Lastly, differences in the IL10 and IL12p40 cytokine levels in men and IL12p40 and IL12p70 cytokine levels in women, are also gender specific biomarkers.

One of our most important findings was that the re-establishment of immunological (Th cell-network) and neurological (no increase of EDSS) homeostasis in treated patient is induced by gender specific cytokine pathways, since the IL6 vector in women and the IFNγ vector in men negatively opposed the respective EDSS vectors (Figure 6a: IDC; 6b: DC). Furthermore, IL6 pathways are re-established: the above mentioned relationship between the production of IL6 and IL4 in men, changes from negative (condition of untreated patients) to positive (condition of healthy subjects). Similarly in women, the relationship between the production of IL6 and IL10 changes from positive (condition of untreated patients) to negative (condition of healthy subjects) (Figures 6a, 6b and 7). Additionally, CD30 pathways are also re-established: we found that IDC and DC from treated male and female patients, compared to IDC and DC from untreated patients, secreted significantly lower sCD30 levels and significantly higher TGFβ1 levels (Table 2). Furthermore the relationship between the DC sCD30 and EDSS production in men, changes from positive (condition of untreated patients) to negative (condition of treated patients) (Figure 6a: IDC; 6b: DC). Likewise in women, the relationship between the IDC sCD30 and EDSS changes from positive (condition of untreated patients) to negative (condition of treated patients) (Figure 6a: IDC; 6b: DC).

**Figure 8:**
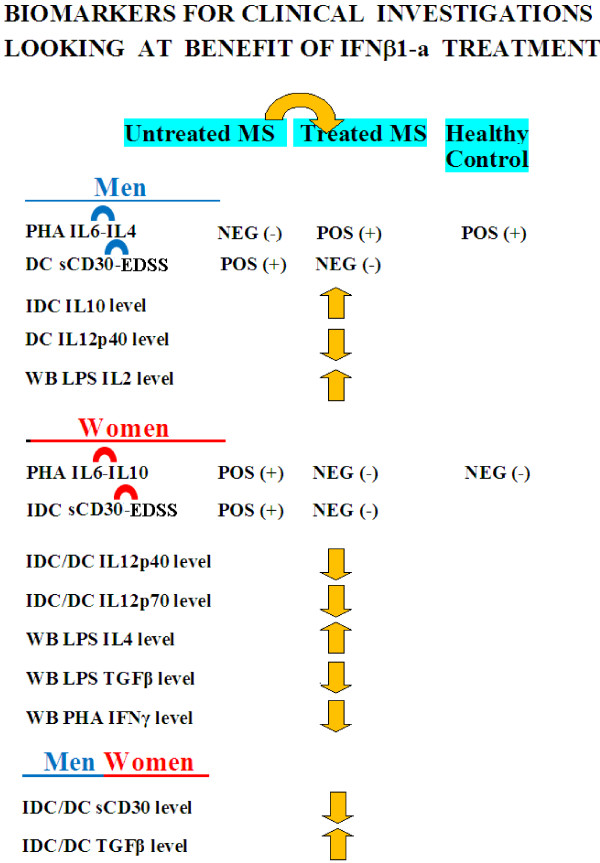
**Gender specific biomarkers for homeostasis in immunological, neurological and therapeutic pathways.** Re-establishment of immunological and neurological homeostasis in treated patients induced by gender specific cytokine pathways, re-establishes the above relationships between the production of IL6 and IL4 (from negative, the condition of untreated patients, to positive, the condition of healthy subjects) in men; similarly in women homeostasis is re-established when the relationship between the production of IL6 and IL10 reverses (from positive, the condition of untreated patients, to negative, the condition of healthy subjects). Additionally, the significance of sCD30 and TGFβ as a dual clinical biomarker was confirmed for both sexes and gender specific new clinical biomarkers were identified.

In a previous study on MS patients [22] we found that the “down- or up-regulation” of sCD30 levels, respectively linked to the “up- or down-regulation” of TGFβ levels, within physiological or pathological ranges, are dual biomarker for homeostasis or imbalance in immunological and neurological pathways and success or failure of IFNβ therapeutic treatment. The results of this study confirm the significance of sCD30 and TGFβ as dual biomarker and identify new gender specific clinical biomarkers (Figure 8). In fact we found that IDC also secreted higher IL10 cytokine levels in men while lower IL12p40 and IL12p70 cytokine levels in women; DC also secreted lower IL12p40 cytokine levels in men while lower IL12p40 and IL12p70 cytokine levels in women (Table 2). Further confirmation also came from the finding that significant differences between untreated and treated patient groups were also found in the network profiles of whole blood Th-cytokine data-driven computational models of disease state (Table 3): a significant increase in LPS-IL2, while a significant increase in LPS-IL4 and decrease in PHA-IFNγ and LPS-TGFβ1 levels were found in men and women profiles respectively, in treated and untreated groups.

### Dual gender-specific biomarkers for homeostasis in immunological, neurological and therapeutic pathways, and translational research for gender-specific drugs

The positive and negative relationships of PHA-cytokines in healthy men and women (Figure 1, p-cytokines) appear to represent gender specific biomarkers for homeostasis in the differentiation of activated T cells (Figure 8).

In fact from Figure 1 it would appear that, in the early evolution of activated T cells (24 h, +PHA culture), the positively linked production of IL6 and IL4, IFNγ and IL10 cytokines are gender specific biomarkers in men, while the negatively linked production of IL6 and IL10 cytokines are gender specific biomarkers in women (Figure 1, p-cytokines). Likewise in the late evolution of activated T cells (72 h, +PHA culture), the positively linked production of IFNγ and IL4 are gender specific biomarkers in men, while IFNγ and IL6 are gender specific biomarkers in women (Figure 1, p-cytokines).

Furthermore (Figure 8), lower sCD30 and higher TGFβ1 levels in cytokine level profiles, is a positive dual gender-common biomarker in clinical investigations looking at IFNβ1a treatment. Gender-specific targets and biomarkers for male and female therapeutic success rates are: i) an increase of IL10 levels from IDC in men, while a fall in IL12p40 and IL12p70 levels from IDC in women; ii) a fall in IL12p40 level from DC in men while a fall in IL12p40 and IL12p70 levels from DC in women; iii) an increase in LPS-IL2 in men, and an increase in LPS-IL4 and a decrease in PHA-IFNγ and LPS-TGFβ1 levels in women, both in whole blood culture (Table 3 and Figure 8).

## Discussion

One of the most important epidemiological risk factors for autoimmune diseases, such as multiple sclerosis, is the female sex. This susceptibility to abnormal autoimmune function has mainly gone unexplained but it is believed that the effects of sex hormones on the immune system may play an important role. The understanding of the physiological and pathological mechanisms of sexual dimorphism in the functioning of the immune system is therefore of considerable importance and may pave the way for new therapeutic strategies.

Autoimmune reactions are determined by auto-reactive T-cells, but are inhibited if Treg cells are present; the experimental removal of Treg cells has been shown to lead to autoimmune reactions [43]. Research has shown that IL6 is critical in preventing the conversion of naive Th cells into Treg cells in vivo by switching Th cell differentiation from Treg to Th17 [44], inhibiting the TGFβ-driven expression of Foxp3 and inducing the expression of ROR-γt transcription factors. Hence, the IL6 cytokine pathway is a nodal point in the shaping of an adaptive immune response and we believe that sexual dimorphism in autoimmune diseases is a result of differing cytokine pathways being involved in the regulation of Th cell network homeostasis.

Hence we focused on sexual dimorphism in relation to changes in the immune response and, in particular, how these changes are related to the homeostasis of the cytokine network and regulation pathways of the Th cell network. In a systems biology study we evaluated healthy subjects and multiple sclerosis patients (IFNβ-treated and untreated) using cytokines, sCD30 and EDSS parameters as biomarkers for Th cell differentiation and neurological deficit.

Our results confirmed our hypothesis, highlighting sexual dimorphism in healthy subjects: we found that IL6 pathways regulate the homeostasis of the Th cell network in women whilst this homeostasis is regulated by IFNγ pathways in men (Figures 1 and 2). This means they could be used as targets and biomarkers for translational research for the development of gender specific therapy. Furthermore, the more significant role of ΙL6 in autoimmune disease with regards to IFNγ explains the greater susceptibility of women to autoimmune disease.

Our finding that immunological alterations in the Treg cell pathways are responsible for the increase of EDSS in both untreated, men and women, MS patients confirms our conclusions (Figure 4). In fact, IL6 together with TGFβ pathways, a dual biomarker for Treg/Th17-network imbalance, were implicated in the EDSS increase in both untreated, men and women groups of MS patients (Figures 3c and 3d: the vectors of TGFβ, IL6 and EDSS are positively related). In IFNβ-treated patients, on the other hand, the IL6 pathway in women but the IFNγ pathway in men were responsible for delaying EDSS (Figure 6a, 6b: the IL6 vector in women, while the IFNγ vector in men negatively opposed the EDSS vector).

In recent studies, looking at the role of sCD30 in immunological and neurological pathways [22], we noted that the down- or up-regulation of sCD30 levels respectively linked to the up- or down-regulation of TGFβ levels, within physiological or pathological ranges, represented a dual biomarker for homeostasis or imbalance in immunological and neurological pathways and hence for the success or failure of IFNβ treatment. On this basis, one of our most important findings was that CD30 pathways are re-established: we found that a fall in the level of sCD30 is linked to an increase in TGFβ levels in both treated patient sex groups compared to untreated patient sex groups (Table 2).

Hence, sCD30 levels linked to TGFβ levels can be used as a dual gender target and biomarker in both sexes to evaluate the success of IFNβ therapy in terms of re-establishment of Th network balance and the delay of the progression of neurological disability (EDSS increase) (Figure 8). Furthermore it is possible to evaluate gender specific success of therapy by looking at the positive linked relationships between cytokine levels of IL6-IL4 and IFNγ-IL10 in men, while the negative one between IL6-IL10 and the positive ones between IL2-IL4, IL6-IFNγ in women (Figure 8).

Overall, our findings underline the need for gender specific drugs that take into account the differing regulation of the immune response whilst ensuring the same result: a physiological homeostasis in the resting state, in the transition to the activation phase and in the return to the resting state. Obviously these regulatory differences do not usually have consequences until pathway alterations occur (Figure 2). In fact, it is well known that in health, in both sexes, the immune system carries out its responses and recovers its initial homeostasis, regardless of differing initial conditions or evolution. If, however, these alterations occur in IFNγ and/or IL6 cytokine pathways, the consequences for men and women, in terms of pathological mechanisms and disease development, are different (Figure 4). The malfunctioning of gender specific pathways not only compromises the homeostasis of the immune response, but may also cause a pathological polarization of T cell subsets specific to each sex: IFNγ supports the development of Th1 functions [28] promoting cell mediated immunity, while IL6 cytokine supports Th2 responses where B lymphocytes are activated and antibody production flourishes. In fact, IFNγ and IL-6 cytokines were initially classified as the cytokines responsible for generating Th1 and Th2 type immune responses respectively. Research in this field, however, has shown that it is not a single cytokine that determines a particular response, but rather the interaction of individual cytokines within a network. Our results suggest that a differing susceptibility and clinical course in MS is caused by different Treg, Th17 and Th9 cell polarization determined by the TGFβ, IL6, IFNγ and IL4 cytokine pathway interactions which vary between men and women (Figure 3 and 4).

These findings are backed up by the results of other researches indicating that there is a reciprocal developmental relationship between Treg, Th17 and Th9 cells because: i) TGFβ triggers the expression of Foxp3 transcription factor in naïve T cells, generating Treg cells, but ii) IL6 inhibits the TGFβ driven expression of Foxp3, and TGF β together with IL-6 induce ROR-gt transcription factor, triggering the developmental program of Th17 cells [31], while ii) IL4 also inhibits TGFβ induction of Foxp3 expression, but TGFβ together with IL4 induce Th9 cells. On the other hand, co-expression of IL-9 and IL-17 was identified as a novel Th17 function in mediating autoimmune tissue destruction [45,46]. In fact, the IL9 receptor complex is constitutively expressed on astrocytes. IL9 induces astrocytes to produce CCL-20 but not other chemokines, including CCL-2, CCL-3, and CXCL-2 [47], suggesting that IL9 induces CCL-20 production by astrocytes to induce the migration of Th17 cells into the CNS. Treg, Th9 and Th17 cells have been shown to be important CD4 T cell subsets in human autoimmune diseases, including rheumatoid arthritis [48] and multiple sclerosis [49]. So, it follows that there is sexual dimorphism in the regulation of the Th cell network. IL6 and IFNγ pathway are the respective male and female pathways used to regulate immune and neurological homeostasis and therefore targets and biomarkers to evaluate gender specific IFNβ therapy in MS.

## Conclusion

Our results confirm that gender dimorphism in autoimmune diseases is a result of sexual dimorphism in the regulation of Th cell network homeostasis in the immune response and the susceptibility of the female sex to abnormal autoimmune function, such as MS, can be attributed to the dominant role of ΙL6. The higher probability of men developing the primary progressive MS form, on the other hand, can be attributed to the role of IFNγ in Th9 cell inhibition. Understanding how these differing pathways lead to disease and how they interfere with the success of current therapies is of utmost importance in translational medicine and physiological treatment.

## Abbreviations

(Th): T-helper; (MS): Multiple sclerosis; (APC): Antigen presenting cell; (IFN): Interferon; (s): Soluble; (EDSS): Expanded disability status scale; (IL): Interleukin; (Treg): T regulatory; (TGF): Transforming growth factor; (FOXP3): Forkhead box P3; (RORgt): Retinoic acid-related orphan receptor gt; (LPS): Lipopolysaccharide; (PHA): Phytohaemagglutinin; (PBMC): Peripheral blood mononuclear cells; (I): Immature; (DC): Dendritic cells; (FcR): Fc receptors; (PCA): Principal component analysis.

## Competing interests

The authors declare that they have no competing interests.

## Authors’ contributions

IC and PP contributed to design the research and performed experiments; RT and AC carried out the neurological examinations of MS patients; TDB contributed to perform experiments; AMB designed the research, performed models, analyzed and interpreted data, wrote the manuscript. All authors read and approved the final manuscript.
